# Species identity and neighbor size surpass the impact of tree species diversity on productivity in experimental broad-leaved tree sapling assemblages under dry and moist conditions

**DOI:** 10.3389/fpls.2015.00857

**Published:** 2015-10-26

**Authors:** Torben Lübbe, Bernhard Schuldt, Christoph Leuschner

**Affiliations:** Plant Ecology and Ecosystems Research, Albrecht von Haller Institute for Plant Sciences, University of GöttingenGöttingen, Germany

**Keywords:** aboveground productivity, belowground productivity, complementarity effect, drought sensitivity, interspecific competition, neighbor effect, selection effect

## Abstract

Species diversity may increase the productivity of tree communities through complementarity (CE) and/or selection effects (SE), but it is not well known how this relationship changes under water limitation. We tested the stress-gradient hypothesis, which predicts that resource use complementarity and facilitation are more important under water-limited conditions. We conducted a growth experiment with saplings of five temperate broad-leaved tree species that were grown in assemblages of variable diversity (1, 3, or 5 species) and species composition under ample and limited water supply to examine effects of species richness and species identity on stand- and tree-level productivity. Special attention was paid to effects of neighbor identity on the growth of target trees in mixture as compared to growth in monoculture. Stand productivity was strongly influenced by species identity while a net biodiversity effect (NE) was significant in the moist treatment (mostly assignable to CE) but of minor importance. The growth performance of some of the species in the mixtures was affected by tree neighborhood characteristics with neighbor size likely being more important than neighbor species identity. Diversity and neighbor identity effects visible in the moist treatment mostly disappeared in the dry treatment, disproving the stress-gradient hypothesis. The mixtures were similarly sensitive to drought-induced growth reduction as the monocultures, which may relate to the decreased CE on growth upon drought in the mixtures.

## Introduction

Recent findings from several biodiversity experiments with planted young trees and observational studies in forests suggest that forest productivity is often enhanced by higher tree diversity (e.g., Zhang et al., 2012; Scherer-Lorenzen, 2014). Contradicting evidence does also exist, however, showing no or even a negative relationship of diversity to forest productivity in diversity experiments (Lang et al., 2012; Grossiord et al., 2013; Li et al., 2014) or in forests (Firn et al., 2007; Szwagrzyk and Gazda, 2007; Jacob et al., 2010; Von Oheimb et al., 2011). Theory predicts that three mechanisms may lead to a positive diversity effect on stand productivity, a selection effect (SE) (the probability of including productive species in the sample increases with increasing species richness), greater complementarity in resource consumption at the stand level, and facilitative interactions that enhance growth (Vandermeer, 1992; Loreau and Hector, 2001). A key process in the diversity–function relationship in forests is competition, which is underlying the selection process in mixed forests, but which is also important for the complementarity effect (CE) as complementary resource use should reduce competition intensity. Increasing diversity should lead to increasingly asymmetric competitive interactions in a stand. Species identity influences stand productivity not only through the traits of the occurring species, but also via neighbor effects on the growth of target trees; the latter effects may be species-specific.

Only few experiments with planted young trees are able to separate between true diversity effects on productivity as caused by resource use complementarity and/or facilitation, and SE, which are driven by the presence of certain species with specific properties (Potvin and Gotelli, 2008; Lang et al., 2012; Grossiord et al., 2013). This is also true for effects of tree neighbor composition on growth. In dependence of their competitive strength, neighbors may decrease or increase the growth of target trees in relation to growth in monoculture. Consequently, these effects should differ between pure stands and mixtures and vary with neighbor species identity (Stoll and Newbery, 2005; Pretzsch and Schütze, 2009; Mölder et al., 2011; Lang et al., 2012). The size and density of neighbors are known as key factors influencing the competitive ability and performance of target plants (e.g., Weiner, 1990). However, their effect has been found difficult to separate from tree identity effects, i.e., neighbor properties other than plant size and density acting on target plants. Several studies showed that neighbor identity effects can be modified or even masked by crowding or tree size effects (Uriarte et al., 2004; Potvin and Dutilleul, 2009; Von Oheimb et al., 2011; Lang et al., 2012).

The interplay between species identity and diversity effects on forest productivity and the relative importance of neighbor effects on tree growth are not well understood. Even less is known about the environmental dependence of these processes on forest ecosystem functioning. The stress-gradient hypothesis applied to forests predicts that resource use complementarity and facilitation are of greater significance in stressful environments (Callaway and Walker, 1997), i.e., in forests exposed to dry, cold, or nutrient-poor conditions, which seems to be supported by empirical studies (e.g., Vilà et al., 2007; Pretzsch et al., 2010; Paquette and Messier, 2011). If positive diversity effects on productivity were indeed larger under stressful conditions, tree species richness could serve to enhance community resistance against environmental hazards. However, it is not well known whether more diverse forests capture resources more rigorously under limiting conditions compared to monocultures (Forrester, 2014). Functional biodiversity research in forests would also benefit from deeper insights into the role of species identity and associated SE on productivity and other ecosystem functions.

Recent comprehensive observational studies along a natural diversity gradient in an old-growth temperate deciduous forest with decreasing abundance of European beech (*Fagus sylvatica* L.) in Hainich National Park (Thuringia, Germany) showed that tree species identity exerted a large influence on various ecosystem functions, while diversity itself seemed to be only of secondary importance (Gebauer et al., 2012; Jacob et al., 2013). Three- and five-species stands were not more productive above-ground than monospecific beech stands (Jacob et al., 2010) but had a higher fine root productivity in ingrowth cores (Meinen et al., 2009). In addition, the stem wood production of beech was higher and its sensitivity to environmental fluctuation lower in more diverse neighborhoods on clay-rich soils, highlighting the role of tree neighborhood effects (Mölder et al., 2011; Mölder and Leuschner, 2014).

Here, we present the results of a tree diversity experiment with potted sapling assemblages, designed to complement the findings obtained from the observational studies in the Hainich mixed forest. The five temperate broad-leaved tree species used in the study (*Fraxinus excelsior* L., *Acer pseudoplatanus* L., *Carpinus betulus* L., *Tilia cordata* L., *F. sylvatica* L.) are also the most abundant species in the Hainich forest; they differ in important morphological and functional traits (Köcher et al., 2009, 2012; Legner et al., 2013). We established three diversity levels (1-, 3-, and 5-species) with all possible monocultures (5) and 3-species combinations (10) and cultivated the plants for 16 months at both ample and water-limited conditions. Study goal was to disentangle the effects of tree diversity and tree species identity on the productivity at the stand level (five trees each) and the tree level under both favorable and resource-limited conditions. Special emphasis was put on neighborhood effects on tree growth and their alteration with increasing diversity.

We tested the hypotheses that (i) stand productivity increases with diversity, but species identity is a more influential factor, (ii) the growth performance of target trees is significantly influenced by the species composition of the neighborhood, (iii) the neighborhood effect is mainly a tree size effect rather than a species identity effect, and (iv) diverse stands reduce their productivity under drought less than monocultures because they reach a higher resource use complementarity.

## Materials and methods

### Experimental design

A replicated diversity experiment with 1- to 2-yr-old saplings of the five common Central European broad-leaved tree species [*F. excelsior* (European ash), *A. pseudoplatanus* (sycamore maple), *C. betulus* (European hornbeam), *T. cordata* (small-leaved lime), and *F. sylvatica* (European beech)] was established in April 2011 in the Experimental Botanical Garden of the University of Göttingen (coordinates: 51°33′ N, 9°57′ E, 177 m a.s.l.) and conducted for two vegetation periods until harvest in August 2012 (duration: 15 months, ~450 days). Five saplings were planted together each in a pot of 0.05 m^3^ volume (height: 0.30 m, diameter: 0.58 m) filled with coarse-grained sand (98% sand, 1.8% silt, 0.2% clay). The plants were arranged systematically at equal distances to each other to expose them to similar competition intensities. We established three diversity levels (1, 3, or 5 species per pot) and grew all five species in monoculture (all five plants of the same species; five types of monocultures), in 3-species mixture (ten possible combinations with three out of five species) and in 5-species mixture (all plants of different species identity). Thus, 16 different species combinations were investigated. The experiment was further conducted with two different soil moisture treatments (moist, dry), which allowed us to test for diversity and species identity effects on growth under optimal and resource-limited conditions. Due to limitations in plant material and work force, the dry treatment could not be carried out with the full set of species combinations used in the moist treatment. The 10 possible 3-species mixtures were reduced in the dry treatment to five representing each species in three different combinations (Table 1). We defined target values of maximal volumetric soil water content (SWC) for the moist (~21%) and the dry treatment (~12%), equivalent to 95%, and 57% of field capacity, respectively. In total, 185 pots with 925 tree individuals were monitored. For details see Lübbe et al. (2015).

**Table 1:** **Design of the experiment with five tree species, three diversity levels (mono, monocultures; mix 3, 3-species mixtures; mix 5, 5-species mixtures) and moist and dry treatments with the number of replicates**.

**Thee diversity**	**Species combination**	**Replication (*****n*****)**	
		**moist**	**dry**
**MONO**			
	*A. pseudoplatanus*	7	7
	*C. betulus*	7	7
	*F. sylvatica*	7	7
	*F. excelsior*	7	7
	*T. cordata*	7	7
**MIX-3**			
	A.p. – C.b. – F.s.	7	
	A.p. – C.b. – F.e	7	6
	A.p. – C.b. – T.c	7	6
	A.p. – F.s. – F.e.	7	6
	A.p. – F.s. – T.c.	7	
	A.p. – F.e. – T.c.	7	
	C.b. – F.s. – F.c.	7	
	C.b. – F.s. – F.e.	7	6
	C.b. – F.e. – T.c.	7	
	F.s. – F.e. – T.c.	7	6
**MIX-5**			
	A.c. – C.b. – F.s.		
	–F.e. – T.c.	8	7

*In the dry 3-species mixtures, only five of the 10 possible combinations were realized. A.p., Acer pseudoplatanus; C.b., Carpinus betulus; F.e., Fraxinus excelsior; F.s., Fagus sylvatica; T.c., Tilia cordata*.

The pots were installed under a light-transmitting roof, which excluded all precipitation. The pots were set up at random position in a grid pattern for minimizing the impact of possible environmental gradients.

During July-September 2011 and May-August 2012, mean SWC content varied between 12 and 20% in the moist and 7 and 12% in the dry treatment. Accordingly, lowest soil matrix potentials reached −84 kPa in the moist and −869 kPa in the dry treatment, respectively (Lübbe et al., 2015). Soil moisture content and the amount of required irrigation water were determined gravimetrically. For details on plant care and soil moisture control see Lübbe et al. (2015). Details on climatic conditions are provided in Figure A1.

### Measurement of productivity, allocation patterns, and plant morphology

The final harvest of all plants took place within a 7-week period in July/August 2012, i.e., up to 16 months after the onset of the experiment. By applying a rotating harvesting scheme, one replicate pot per treatment and species combination was collected every week, thereby avoiding different experimental durations of the treatments. The roots were washed out from the substrate under flowing water. Shoot length (L_Shoot_) and maximum root length (L_Root_) were determined and the stem diameter at ground level was measured in two directions perpendicular to each other for calculating basal area (BA). Leaf, stem and root mass were oven-dried (70°C, 72 h) and weighed at a precision of 10 mg. The specific leaf area (SLA) of fully expanded leaves in the upper crown was determined for a subset of trees using WinFolia software (Régent, Quebec, Canada); it served for calculating the total leaf area (LA) of the trees. Besides metrics related to tree size, biomass, and biomass partitioning, we calculated the root-to-shoot ratio (RS) and the relative increment in BA (BAI), shoot length (LI_Shoot_), and root length (LI_Root_) for the entire growth period of 450 d by subtracting initial from final size or biomass (initial plant metrics are given in Table A1). Furthermore, relative growth rates (RGRs) were calculated considering aboveground, below-ground and total biomass (RGR, in g g^−1^ 450 d^−1^). Growth was measured with the aim (i) to compare the productivity of a tree assemblage in a pot among different species combinations, diversity levels, and soil moisture levels, and (ii) to analyze the productivity of the five species on the tree individual level in its dependence on diversity, neighborhood, and soil moisture level. Net biodiversity effects (NE), selection effects (SE), and complementarity effects (CE) on stand productivity were calculated after Loreau and Hector (2001) using Equation (1):

(1) $$\begin{array}{l} NE=CE+SE=N\;\bar{△RY}\;\bar{Y_{M}}+N\;cov\;\left(△RY,Y_{M}\right) \end{array}$$

where *N* is the number of species in mixture and Δ*RY* is the difference between observed and expected relative yield (the latter being derived from the species' relative abundance in the mixture upon planting). *Y*_*M*_ is the yield of a species in monoculture. Horizontal bars above terms symbolize average values across the species in mixture. *COV* is the covariance of the two variables in parentheses. Neighborhood effects on the growth performance of a target species were investigated in the 3-species mixtures, where for every species all six possible neighborhood constellations with the four other species were realized in the moist treatment. In the dry treatment, only three of the six possible combinations were available (Table 1). We calculated the competitive ability index (CA) after Grace (1995), which compares the growth performance of a target species in mixture with that in monoculture (Equation 2).

(2) $$\begin{array}{l} CA=\frac{\left(RGR_{mix}-RGR_{mono}\right)}{RGR_{mono}} \end{array}$$

We did this for all six neighborhood constellations of a species in the 3-species mixtures (moist treatment; only three in the dry treatment). To disentangle the effect of the each two neighbors in the 3-species mixtures, we also calculated the CA of a target species for all species combinations where one of the four possible neighbors was present.

### Statistical analysis

To avoid pseudo-replication, we used the pots as replicate units in samples consisting of the different individuals of a species. We thus averaged over all individuals of a species in a pot. Statistical analyses were done with R software, version 3.0.0 (R Development Core Team, 2014). We conducted Two-way ANOVAs to test for effects of the factors species composition (Type II SS considering incomplete data) or tree diversity (Type III SS for unbalanced designs) in assumed interaction with soil moisture treatment on parameters characterizing productivity and biomass partitioning at the pot level (*car* package). For the additive partitioning procedure of biodiversity effects, grand means of the NE, SE, and CE were tested against zero by one-sample *t*-tests. We further tested the effect size of species richness (three or five species; *t*-test) or species composition (ANOVA) on the variance of the three diversity effects. At the tree-individual level, Three-way ANOVAs were conducted for analyzing effects of species identity, diversity level, and moisture treatment on various growth-related parameters. The effect of the neighbor constellation in 3-species mixtures on the RGR and CA of the five species was tested individually by One-way ANOVAs in the moist and dry treatment. To test for the influence of certain neighbor species on the RGR_total_ and CA of a target species in 3-species mixtures, we applied generalized linear models (glm), where the presence of heterospecific neighbors was introduced through dummy variables (yes/no). For separating between effects of neighbor identity and crowding on the growth of target species in the mixed pots, we further conducted ANCOVA analyses with the species composition of the neighborhood as predictor variable and several parameters characterizing the size of the neighbors (biomass, LA, shoot length, root length) introduced separately as co-variables. The residuals of all models were tested for violation of the normality (Shapiro-Wilk test) and homoscedasticity assumptions (Levene's test). Multiple comparisons among the means of different species, species combinations or diversity levels were performed with Tukey contrasts (*glht (), multcomp* package). Pairwise comparisons among the two moisture treatments were done with Student's *t*-test, Welch's *t*-test, or the Mann-Whitney U-test, depending on data structure.

## Results

### Stand productivity and biomass partitioning

While average phytomass production and RGR of the sapling assemblages tended to increase slightly from the monospecific to the 3-species and the 5-species mixtures for most studied parameters (phytomass, LA, BA, RGR_above_, RGR_below_, RGR_total_), the increase was significant only for LA (in the moist treatment), and LI_Root_ (Table 2). In contrast, L_Shoot_ and root:shoot ratio (RS) were not affected. These results are consistent with those from Two-way ANOVA, which showed a significant diversity effect only for LA [*F*_(2, 183)_ = 3.78, *p* < 0.05] but not for the other productivity parameters including RGR_total_ [*F*_(2, 183)_ = 1.10, *p* > 0.10].

**Table 2:** **Various parameters characterizing productivity and plant-internal biomass partitioning (pot-level data: 5 plants each) averaged over the three diversity levels in the moist and dry treatments**.

**Moisture treatment**	**Diversity level**	**No. of replicates (*n*)**	**Phytomass (g)**	**RS (g g^−1^)**	**LA (m^2^)**	**BA (cm^2^)**
Moist	mono	35	511.90 ± 27.88 a	1.08 ± 0.06 a	1.46 ± 0.07 a	10.32 ± 0.81 a
	mix3	70	547.70 ± 15.37 a	1.12 ± 003 a	1.65 ± 0.04 b	10.99 ± 0.31 a
	mix5	8	554.88 ± 42.04 a	1.11 ± 0.06 a	1.55 ± 0.08 ab	11.81 ± 0.68 a
Dry	mono	35	425.70 ± 22.70 a^*^	1.11 ± 0.06 a	1.29 ± 0.07 a^◦^	8.60 ± 0.70 a^*^
	mix3	30	434.59 ± 13.69 a^***^	1.10 ± 0.04 a	1.41 ± 0.05 a^***^	8.75 ± 0.38 a^***^
	mix5	7	445.49 ± 23.88 a	1.02 ± 0.02 a	1.45 ± 0.05 a	9.36 ± 0.29 a^**^
**Moisture treatment**	**Diversity level**	**No. of replicates (*****n*****)**	**L_Shoot_ (cm)**	**L_Root_ (cm)**	**LI_Shoot_ (%)**	**LI_Root_ (%)**
Moist	mono	35	100.37 ± 4.06 a	70.94 ± 3.27 a	121.51 ± 12.18 a	184.89 ± 8.37 a
	mix3	70	97.87 ± 1.36 a	76.67 ± 167 a	108.46 ± 4.13 a	205.78 ± 5.43 b
	mix5	8	98.45 ± 3.45 a	82.75 ± 3.43 a	107.36 ± 7.28 a	230.15 ± 13.70 b
Dry	mono	35	86.62 ± 2.62 a ^**^	64.66 ± 2.48 a	90.34 ± 8.79 a^*^	162.98 ± 9.39 a^*^
	mix3	30	85.44 ± 1.72 a^***^	65.75 ± 1.48 a^***^	82.78 ± 5.98 a^***^	164.64 ± 6.45 a^***^
	mix5	7	87.98 ± 1.18 a^*^	69.52 ± 3.87 a^*^	85.33 ± 2.49 a^*^	177.36 ± 15.43 a^*^
**Moisture treatment**	**Diversity level**	**No. of replicates (*****n*****)**	**BAI (%)**	**RGR_above_**	**RGR_below_**	**RGR_total_**
Moist	mono	35	337.15 ± 22.90 a	6.10 ± 0.43 a	3.77 ± 0.24 a	4.68 ± 0.28 a
	mix3	70	329.00 ± 11.76 a	6.38 ± 0.19 a	4.23 ± 0.18 a	5.11 ± 0.17 a
	mix5	8	341.58 ± 25.37 a	6.44 ± 0.52 a	4.33 ± 0.47 a	5.19 ± 0.47 a
Dry	mono	35	258.73 ± 16.61 a^**^	4.70 ± 0.30 a ^**^	3.06 ± 0.20 a^*^	3.70 ± 0.21 a^**^
	mix3	30	240.75 ± 11.02 a^***^	4.96 ± 0.22 a^***^	3.14 ± 0.13 a^***^	3.85 ± 0.15 a^***^
	mix5	7	250.03 ± 11.03 a^**^	5.22 ± 0.33 a^◦^	3.11 ± 0.22 a^*^	3.97 ± 0.27 a^*^

For phytomass, leaf area (LA) and basal area (BA), cumulative values for the five plants are given, for root:shoot ratio (RS), shoot length (L_shoot_), root length (L_root_), shoot and root length increment (LI_shoot_, LI_root_, in percent of initial value), basal area increment (BAI, in percent), and RGR, averages over the five plants are presented. Relative growth rates (RGR) are given in g g^−1^ 450 d^−1^. Different small letters indicate significant differences between diversity levels (p < 0.05); asterisks in the dry treatment indicate significant differences between moisture treatments in a diversity level (

◦ *p < 0.10*;

* *p < 0.05*;

** *p < 0.01*;

*** *p < 0.001)*.

*Note different no. of replicates in the diversity levels*.

**Figure 1:**
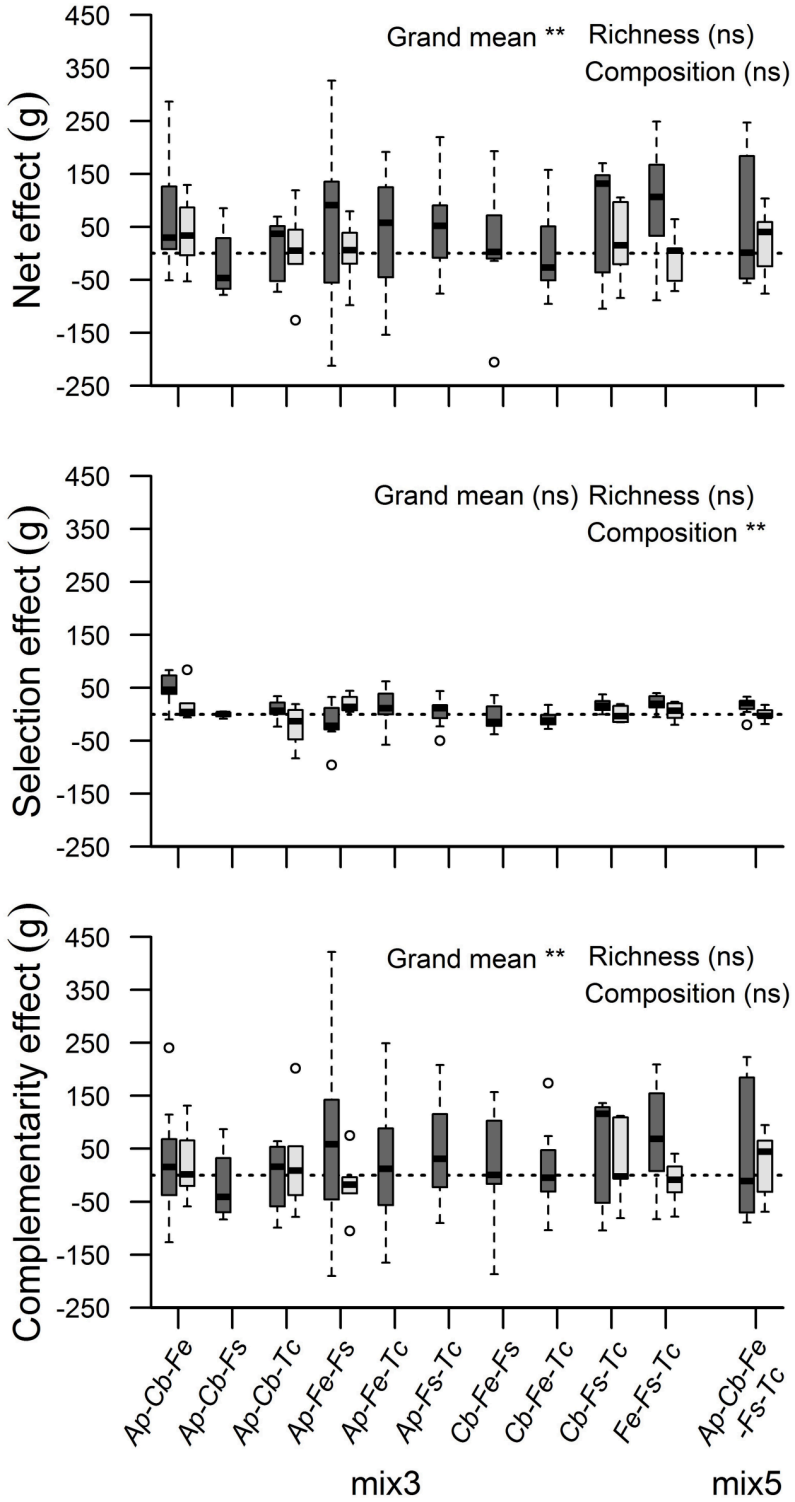
**Additive partitioning of biodiversity effects on accumulated biomass of mixed tree assemblages: Net diversity effect, selection effect, and complementarity effect in their dependence on species richness (three vs. five species) and species composition**. Asterisks indicate significant effects for the moist treatment (dark boxes; ^**^p < 0.01; ns, non-significant). In the dry treatment, no significant effects were detected (bright boxes). Circles above and below boxplots show outlier values.

Additive partitioning of biodiversity effects after Loreau and Hector (2001) showed, for the moist treatment only, a significant NE on biomass (*t* = 3.87, *p* < 0.01), which was mainly due to a positive CE (*t* = 3.67, *p* < 0.01; Figure 1). Across all 11 mixtures, a significant SE on biomass production was not detected. CE, SE and NE were not influenced by species richness (3-species vs. 5-species mixtures), and the species composition of the mixtures influenced only the size of the SE significantly (*F* = 3.34, *p* < 0.01). Similar patterns for NE and CE were detected for various other growth-related parameters with strongest effects for LA and L_Root_ (Table A3). Significant SE co-occurred in case of below-ground biomass, LA and BA.

All 12 productivity-related parameters except RS were significantly affected by the moisture treatment (Table 2). In contrast to the moist treatment, significant NE and CE occurred in the dry treatment only by exception (above-ground biomass and LA, Table A3). The reduction in RGR from the moist to the dry treatment tended to increase with diversity and it was more conspicuous in root growth than in shoot growth (RGR_below_: 19, 26, and 28% reduction in the monospecific, mix 3 and mix 5 category, respectively).

### Species identity effects on stand productivity and biomass partitioning

**Figure 2:**
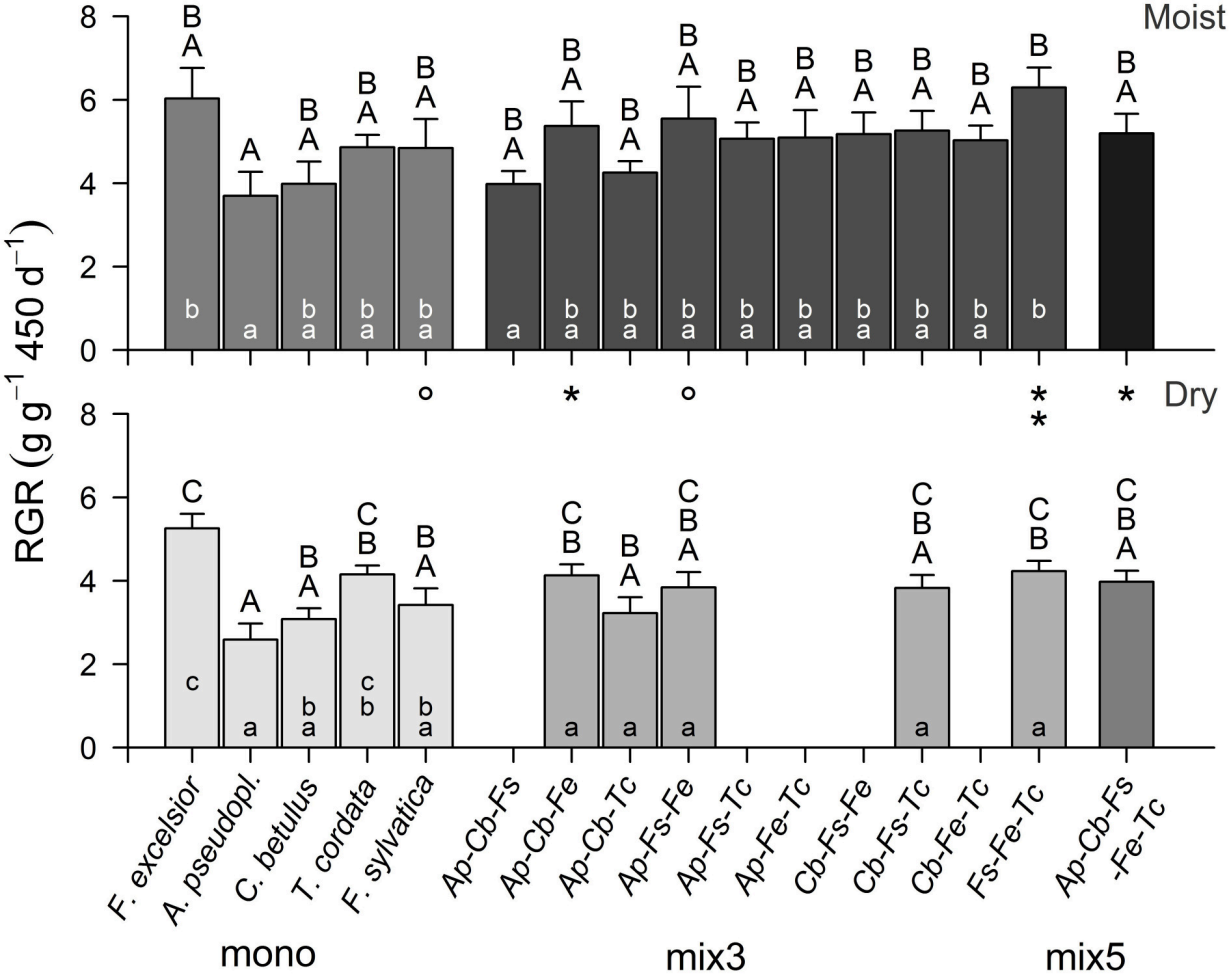
**Average relative growth rate (RGR; above- and below-ground) of tree assemblages differing in species composition and diversity in the moist (upper panel) and dry (lower panel) treatment (mean ± SE of 6–8 replicate pots)**. Different capital letters indicate significant differences (*p* < 0.05) between the species combinations in the full sample (moist: 16, dry: 11 combinations), different small letters indicate significant differences between the species combinations within a diversity level. Asterisks indicate significant differences between the moisture treatments for a species combination (°*p* < 0.10; ^*^p < 0.05; ^**^p < 0.01). For species abbreviations see Table 1.

Comparing the pot-level productivity of the 16 (moist treatment) or 11 species combinations (dry treatment) with Two-way ANOVA revealed highly significant effects of the species combination [F_(15, 169)_ = 3.75, *p* < 0.001] and of the moisture treatment [*F*_(1, 183)_ = 38.28, *p* < 0.001] on RGR_total_. The largest productivity differences existed among the five monocultures (3.7–6.0 g g^−1^ 450 d^−1^ in the moist treatment, 2.6–5.3 g g^−1^ 450 d^−1^ in the dry treatment) with highest RGR_total_ in *F. excelsior* and lowest in *A. pseudoplatanus* (difference significant in both treatments; Figure 2). Except for one 3-species mixture (*Fagus-Fraxinus-Tilia*: 6.3 g g^−1^ 450 d^−1^ in the moist treatment), the RGR of all 3- and 5-species mixtures remained in the productivity range set by the five monocultures, and transgressive over-yielding was restricted to this single mixture.

Variation in pot-level RGR_total_ among the different mixtures was smaller in the dry than in the moist treatment, and significantly different productivities of the various 3-species combinations appeared only in the moist treatment.

### Growth of the five species as dependent on neighborhood diversity and composition

The individual-based RGR_total_ analysis allows comparing the growth performance of the species in defined neighborhood constellations. Three-way ANOVA indicated for all growth-related parameters highly significant effects of species identity [RGR_total_: *F*_(4, 420)_ = 20.30, *p* < 0.001] and also of the moisture treatment [RGR_total_: *F*_(1, 423)_ = 21.57, *p* < 0.001], except for RS. Diversity effects were significant only for L_Root_ [F_(2, 422)_ = 8.17, *p* < 0.001] and LI_Root_ [*F*_(2, 422)_ = 8.53, *p* < 0.001].

**Figure 3:**
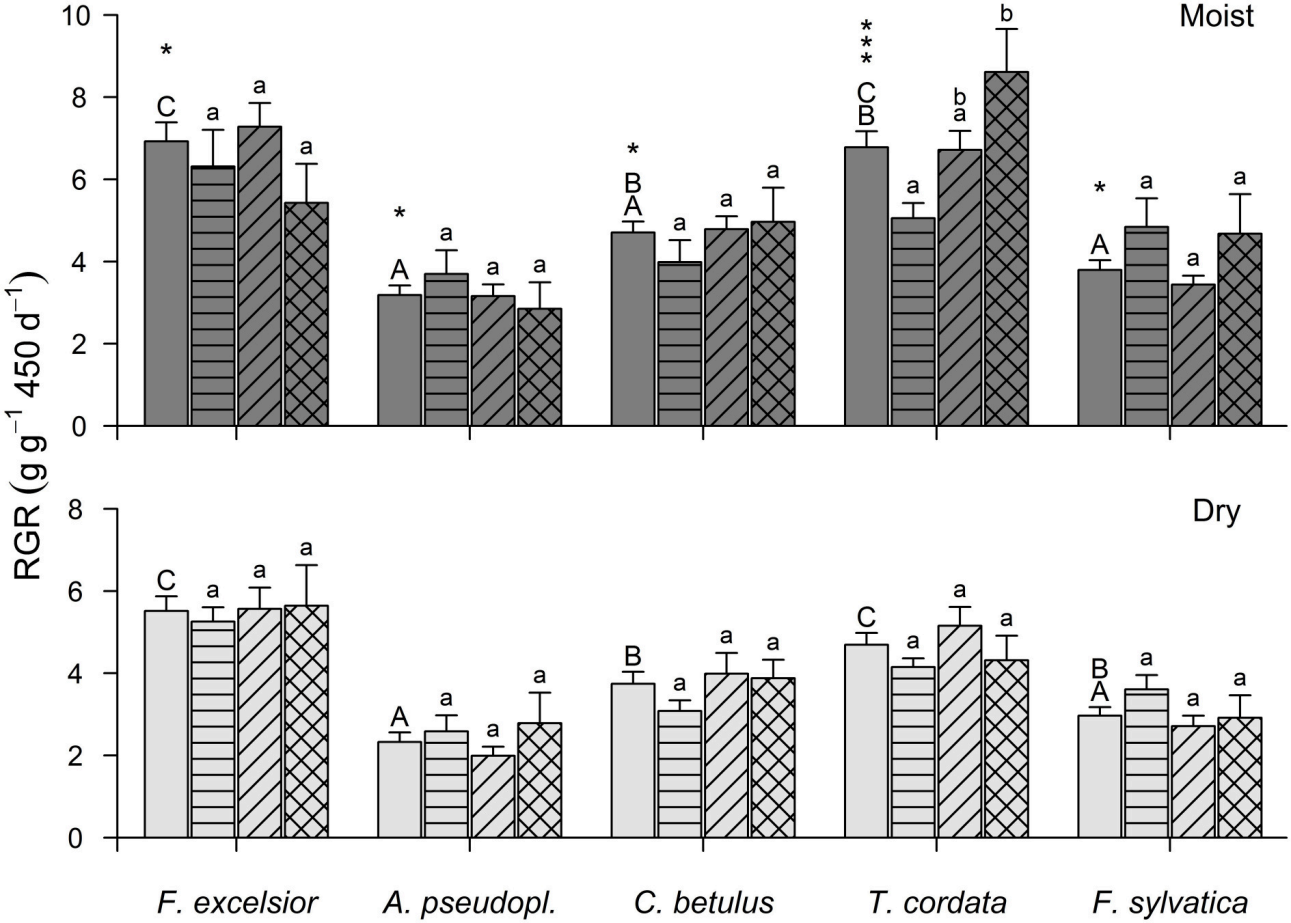
**Relative growth rate (above- and below-ground) of the five species in the moist (upper panel) and dry treatment (lower panel) in monoculture (second bar of a group), 3-species mixture (3^rd^ bar), 5-species mixture (4^th^ bar), and as average of all constellations (first bar, no hatching) (means ± SE)**. Different capital letters indicate significantly different species averages (*p* < 0.05), different small letters significant differences between the three diversity levels within a species. The number of asterisks gives the level of significance for the growth reduction from the moist to the dry treatment of a species (^*^p < 0.05; ^***^p < 0.001).

When all individuals of a species from all species combinations were pooled in the analysis, productivity (RGR_total_) decreased in the sequence *Fraxinus* > *Tilia* > *Carpinus* > *Fagus* > *Acer* in the moist and the dry treatment (Figure 3: first bars of the species blocs). For the other productivity parameters, the species ranking differed in some cases (Table A2).

When comparing a species' RGR_total_ in monoculture, 3-species mixture, and 5-species mixture (Figure 3), RGR_total_ of *T. cordata* was significantly higher in 5-species mixture than in monoculture (5.05, 6.71, and 8.61 g g^−1^ 450 d^−1^ in 1-, 3-, and 5-species assemblages), which was reflected in the significant increase in LA of *Tilia* plants from 1- to 3-species assemblages (Table A7). A non-significant tendency for higher growth rates with increasing diversity was also observed in *C. betulus* (Table A6), while *F. sylvatica, A. pseudoplatanus* and *F. excelsior* (Table A4) showed no productivity trend across the three diversity levels. However, *A. pseudoplatanus* increased both L_Root_ and LI_Root_ in 5-species mixture compared to the monoculture (moist treatment), but decreased RS in 5-species mixture in the dry treatment (Table A5). In contrast, *F. sylvatica* saplings tended to grow better in monoculture than in 3-species mixtures, which was also visible in higher LA, BA, RGR_above_, and a smaller RS (Table A8).

In *T. cordata*, the drought-induced reduction in RGR_total_ increased with diversity (monoculture: −18%, 3-species mixtures: −23%, 5-species mixtures: −50%). For *F. excelsior* (−17, −24, −4%), *A. pseudoplatanus* (−30, −37, −2%), *C. betulus* (−23, −17, −22%), and *F. sylvatica* (−26, −21, −38%), no consistent trends with increasing diversity were visible. In the 5-species mixture, *A. pseudoplatanus* and *F. excelsior* reduced growth only marginally compared to the moist treatment, while *F. sylvatica* and *T. cordata* suffered larger reductions.

### Importance of neighbor species identity

**Figure 4:**
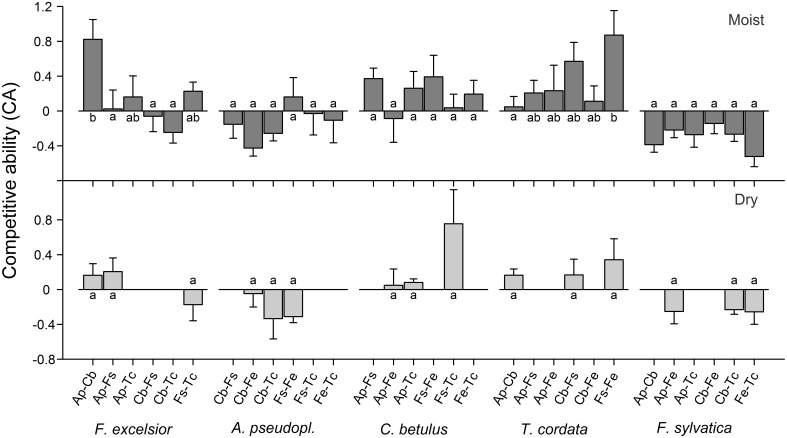
**Competitive ability (expressed as CA index) of the five species when grown in six (moist treatment) or three (dry treatment) different 3-species neighborhood constellations (means ± SE of 6–7 replicate pots)**. For species abbreviations see Table 1. Different small letters indicate significant (*p* < 0.05) differences in CA of the target species between different neighborhood constellations. A positive CA indicates better growth in mixture than in monoculture.

Analysis of variance indicated significant neighborhood effects on the growth response of target species. The superior growth of *F. excelsior* and *T. cordata* in certain 3-species constellations of the moist treatment is reflected in significantly higher CA of the target species in the respective mixtures [Figure 4; ANOVA: *F*_(5, 35)_ = 3.72, *p* < 0.01 for *F. excelsior*; *F*_(5, 34)_ = 2.24, *p* < 0.1 for *T. cordata*]. RGR_total_ of *F. excelsior* was remarkably high in coexistence with *Acer* and *Carpinus* (Figure A2, upper panel) in the moist treatment and the corresponding CA indices were significantly higher than for mixtures with *Acer*—*Fagus, Carpinus*—*Fagus*, and also *Carpinus*—*Tilia* (Figure 4). However, the outstanding performance of *F. excelsior* in combination with *Acer*—*Carpinus* was not observed under dry conditions (Figure 4, lower panel). All CA scores for *T. cordata* were positive indicating better growth in mixture than monoculture with highest values for the coexistence with *Fagus*—*Fraxinus*. In contrast, *F. sylvatica* reached highest growth rates in monoculture resulting in negative CA scores across all heterospecific constellations. Species-specific neighbor effects were less important in the dry treatment. The RGR of *C. betulus* was higher in monoculture than in mixture with *Fagus*—*Tilia* (Figure A2, lower panel), but the CA scores of the different 3-species constellations did not differ (Figure 4).

**Figure 5:**
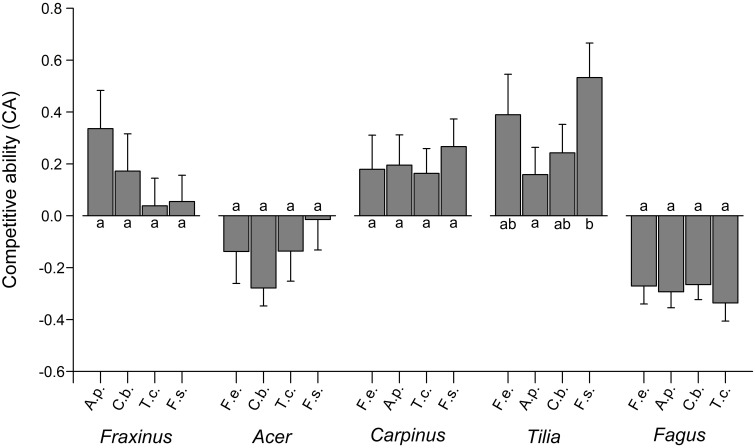
**Competitive ability of the five species in the moist treatment when grown in neighborhood of the respective four other species (means ± SE of 21 neighborhood replicates)**. CA was calculated by pooling the each three 3-species neighborhood constellations in which that neighbor species occurred. Different small letters indicate significant differences (*p* < 0.05) in CA between neighbor constellations of a target species. A.p., *Acer pseudoplatanus*; C.b., *Carpinus betulus*; F.e., *Fraxinus excelsior*; F.s., *Fagus sylvatica*; T.c., *Tilia cordata*.

The explicit analysis of pairwise neighbor interactions on the growth performance of target species in the moist treatment showed *A. pseudoplatanus* and *F. sylvatica* to grow fastest in conspecific neighborhood (negative CA scores; Figure 5; GLM, *glht*), while *F. excelsior, C. betulus*, and *T. cordata* performed better in mixture. Three of the five species did not show significantly different competitive abilities in response to different neighbor species. Only *T. cordata* achieved a significantly higher CA score in neighborhood to *Fagus* than in vicinity to *Acer* (*p* < 0.05). *F. excelsior* showed highest CA scores in coexistence with *Acer*, which tended to be higher than the scores for *Tilia* or *Fagus* as neighbors (*p* < 0.10).

### The importance of neighbor size for competitive interactions

Effects of neighbor size on the CA score of the target species were tested by introducing either neighbor biomass, LA or plant size as co-variable in ANCOVA runs (Table 3). For *F. excelsior* and *C. betulus* in the moist treatment, the models explaining CA were significantly improved when the neighbor's LA was included as co-variable while the interaction term of biomass × neighborhood species composition was the most important covariate for *T. cordata*. The species identity of the neighbors (factor SpecComp) was, however, only influential for the CA of *F. excelsior* (secondary to LA) and *T. cordata*, where it was the dominant factor. In the two species with negative CA scores in interspecific interaction (*F. sylvatica* and *A. pseudoplatanus*), variation neither in neighbor size nor neighbor species identity influenced CA. In the dry treatment, neighbor size effects on productivity were much smaller (significant effect of LA in *T. cordata*, marginally significant effect in *A. pseudoplatanus*) (Table 3).

**Table 3:** **ANCOVA results for the five species on the dependence of competitive ability index (CA) on the predictor variable species composition of the neighborhood (six or three constellations in the moist or dry treatments, respectively) and the most influential parameter characterizing neighbor plant size (leaf area LA, basal area BA, or biomass) as covariate**.

**Species**	**Moist treatment**					**Dry treatment**				
	**Predictor**	***df***	***SS***	***F***	***p***	**Predictor**	***df***	***SS***	***F***	***p***
*F. excelsior*	LA	1	3.18	**17.76**	<**0.001**	LA	1	0.45	2.84	0.116
	SpecComp	5	0.39	**2.18**	**0.081**	SpecComp	2	0.10	0.30	0.743
	Error	32	0.18			Error	13	2.08		
*A. pseudopl*	LA	1	0.65	2.70	0.111	LA	1	0.30	**3.57**	**0.085**
	SpecComp	5	0.66	0.55	0.736	SpecComp	2	0.30	1.78	0.214
	Error	32	7.68			Error	11	0.92		
*C. betulus*	LA	1	1.86	**8.14**	**0.007**	LA	1	0.22	0.56	0.469
	SpecComp	5	0.62	0.54	0.744	SpecComp	2	1.43	1.87	0.201
	Error	34	7.76			Error	11	4.20		
*T. cordata*	Biomass	1	0.19	0.84	0.366	LA	1	0.92	**6.02**	**0.032**
	SpecComp	5	3.70	**3.34**	**0.018**	SpecComp	2	0.00	0.00	0.999
	Bm × SpecComp	5	3.11	**2.81**	**0.036**	Error	11	1.69		
	Error	27	5.79							
*F. sylvatica*	BA	1	0.17	2.16	0.151	LA	1	0.08	1.09	0.315
	SpecComp	5	0.39	0.97	0.448	SpecComp	2	0.32	2.04	0.170
	Error	33	2.63			Error	13	1.01		

*Bold F and p-values highlight significant effects on CA scores (p < 0.10)*.

## Discussion

### Tree diversity and identity effects on productivity

We found a significant NE on total (above- and below-ground) biomass production and growth-determining parameters such as LA in the moist treatment in support of our first hypothesis. Additive partitioning of biodiversity effects after Loreau and Hector (2001) showed that the diversity effect was mainly caused by a CE and not by a SE; the latter refers to a replacement process in which more productive species achieve dominance in the assemblage. This result meets the assumptions for an experiment with tree saplings because a positive SE could only result from canopy expansion of the more productive species, but not from competition-induced alteration of species abundances in the assemblages, as may take place in communities of more short-lived plants.

The resulting net diversity effect increased RGR_total_ in the mixtures by ~10% compared to the average of the monocultures and thus was relatively small. Moreover, a productivity increase occurred only from the monospecific to the 3-species mixtures but not from the 3- to the 5-species mixture. Thus, a diversity increase from one to three species seems to enhance resource use complementarity, but not a further diversity increase from three to five species. This matches the stand transpiration data from this experiment, which show a comparable net diversity effect on water consumption but no difference in transpiration rate between 3-species and 5-species mixtures (Lübbe et al., 2015). Due to the large contribution of water spending species (*F. excelsior* and *T. cordata*) to stand transpiration in the mixed tree assemblages in the moist treatment, the net diversity effect was interpreted mainly as a SE. The observed LA increase with diversity, which is a main determinant of plant water loss, was assigned to both complementarity and SE (Table A3). In contrast to earlier studies (e.g., Forrester et al., 2010), water use efficiency of productivity was not different between the diversity levels (Table A9), i.e., the productivity increase was not greater than the transpiration increase with growing species diversity.

The small diversity effect in our experiment might in part be a consequence of the young age of the saplings and the short duration of the experiment. Complementarity in resource use could increase with the development of structurally more complex canopies and root systems, and the manifestation of a substantial SE in tree assemblages might take years or decades. A meta-analysis of plant diversity experiments indeed found that CE on productivity increase over time (Cardinale et al., 2007). However, diversity effects on forest productivity do not seem to be a universal phenomenon (Forrester, 2014). Diversity experiments with planted trees produced mixed results with either positive (e.g., Erskine et al., 2006; Healy et al., 2008) or lacking diversity effects on productivity or biomass (e.g., Nguyen et al., 2012; Grossiord et al., 2013). Further, a sapling experiment with tropical tree species also did not show diversity effects on tree growth (Lang et al., 2012; Li et al., 2014), even though positive interactions were observed.

Various explanations for only small or lacking diversity effects on stand productivity have been proposed including a low potential for growth stimulation under non-limiting conditions, young tree age, and not fully developed tree interactions, low species numbers, and more or less symmetric competition due to missing functional differentiation among the tree species (Von Oheimb et al., 2011; Lang et al., 2012; Grossiord et al., 2013; Li et al., 2014). Niche differentiation certainly requires the presence of species with sufficient functional dissimilarity as given for instance in case of *F. sylvatica* and *Picea abies* (Pretzsch and Schütze, 2009) or *Eucalyptus globulus* and *Acacia mearnsii* (Forrester et al., 2004), for which complementary resource use and overyielding were observed. Our five broad-leaved species differ in important morphological and physiological traits, but they are functionally more similar than these species pairs, in particular at young age.

The most and the least productive monocultures (*F. excelsior* and *A. pseudoplatanus*) differed nearly by a factor of two in their biomass production in the moist treatment. Similarly large interspecific differences were found for the water consumption of the trees, as the most productive species also transpired most (Lübbe et al., 2015). The majority of other tree diversity experiments also reported a prominent tree identity effect on productivity (e.g., Lang et al., 2012; Grossiord et al., 2013). Our experimental results match observations in the Hainich mixed forest in that species identity was much more influential than diversity. However, the diversity effect on above-ground productivity in the sapling experiment, even though weak, was not detected in the mature stands with 1, 3, or 5 species (Jacob et al., 2010).

### Is the neighbor identity effect mainly a size effect?

Loreau and Hector (2001) quantified the SE by the covariance between the monoculture yield of the species and the change in relative yield of the species in the mixtures. Species that profit from the mixture will expand their canopies and root systems at the expense of inferior species and will eventually dominate the mixture by numbers. Our detailed analysis of neighborhood effects on the species' growth in mixture and monoculture allows insights into the mechanisms underlying selection and species identity effects on productivity. Accordingly, the large observed variation in productivity among the different mixture types is only in part caused by the species constellations and species-specific differences in yield; specific neighbor effects (positive or negative) on the productivity of a target species in mixture add to the variation in yield, thus supporting our second hypothesis. This result is in accordance with other studies demonstrating effects of neighborhood composition on tree growth (Massey et al., 2006; Mölder et al., 2011; Von Oheimb et al., 2011; Lang et al., 2012). Most neighborhood interactions in our study were markedly asymmetric as has been found for other tree mixtures as well (Canham et al., 2004, 2006; Potvin and Dutilleul, 2009; Mölder and Leuschner, 2014).

We found considerable differences in the CA of the five species; the species' CA scores depended largely on neighbor identity. While the fast-growing species generally were better competitors in mixture, slower-growing species (*A. pseudoplatanus* and *F. sylvatica*) were inferior competitors. Fast-growing species (in particular *F. excelsior* and *T. cordata*) were more sensitive to the specificity of the neighborhood constellation than the less productive trees.

A neighbor's tree height and biomass are properties likely influencing the growth of a target species, as these attributes typically correlate with the consumption of light, water and nutrients. Our ANCOVA results indicate that neighbor size is a dominant factor, supporting our third hypothesis. In four of the five species (moist or dry treatment), determinants of light interception and canopy space occupation (LA or biomass) were detected as influential variables affecting the neighbor's CA. Due to fixed plant numbers and distances in the pots, differences in plant size are the main determinant of variation in neighbor crowding. The dominant effect of neighbor size on the growth of target trees is in agreement with results obtained in other tree mixing studies (Uriarte et al., 2004; Potvin and Dutilleul, 2009), which found a larger effect of crowding than neighbor species identity. Our results meet the expectation that neighbor effects on the target tree's growth rate are mainly resource depletion effects controlled by the size of the neighbors, while traits unrelated to size (leaf and root physiological properties, direct chemical and mechanical interactions, indirect biotic interactions, etc.) must be of secondary importance.

### No support for the resource gradient hypothesis

From the resource gradient hypothesis, we had expected stronger resource CE in the dry than the moist treatment and a less pronounced growth decline in the mixtures than the monocultures (He et al., 2013; Forrester, 2014). However, we obtained no clear indication that more diverse stands were more resistant against drought-induced productivity reduction, disproving our fourth hypothesis. This finding is in agreement with the results of a quantification of stand water consumption in our experiment revealing a smaller net diversity effect with respect to transpiration in the dry than in the moist treatment (Lübbe et al., 2015). It also concurs with findings on radial growth in mixed coniferous mountain forests, in which species composition, but not species richness, determined community resistance against drought (DeClerck et al., 2006). In fact, species richness may increase drought exposure in mixed forests when more diverse stands exploit soil water reserves more completely than monospecific stands do (e.g., Grossiord et al., 2014). Beneficial effects of mixed stands with respect to drought resistance have been demonstrated in the form of reduced drought sensitivity of growth in certain tree species (Lebourgeois et al., 2013; Pretzsch et al., 2013; Mölder and Leuschner, 2014). In our study, none of the species showed clear improvement in growth performance in mixture than in monoculture in the dry treatment. The lacking CE with respect to transpiration (Lübbe et al., 2015) and growth in the mixtures of the dry treatment might also be related to the restrictions set by a pot trial, when limited soil volume does not allow for distinct root space partitioning. In the moist treatment of our experiment, in contrast, canopy space partitioning between different species likely has taken place which may have reduced competition for light. This would fit to the prediction of reduced competition for light driving mixture effects in stands with high resource supply (Forrester, 2014), matching findings from other tree diversity experiments (Potvin and Dutilleul, 2009; Lang et al., 2012).

## Conclusions

This sapling study was conducted in conjunction with an observational study in an old-growth mixed forest containing the same species composition. The setting allows some careful extrapolation of the experimental results to real world systems. A CE on productivity existed but it was relatively small and less influential than species identity. Moreover, neighbor effects were found to strongly determine the individual growth performance of tree saplings.

Under drought, the observed CE was smaller than for ample water supply. Contradicting the insurance hypothesis of biodiversity, diverse tree assemblages showed no higher resistance to drought than monocultures. Future biodiversity experiments with trees should search for both positive and negative diversity effects in other water-limited mixed stands and assess the evidence for the proposed insurance function of tree diversity in forests under drought.

### Conflict of interest statement

The authors declare that the research was conducted in the absence of any commercial or financial relationships that could be construed as a potential conflict of interest.
