# Supplementary material for: Species identity and neighbor size surpass the impact of tree species diversity on productivity in experimental broad-leaved tree sapling assemblages under dry and moist conditions
Source: Front Plant Sci. 2015 Oct 26;6:857. doi: 10.3389/fpls.2015.00857 (PMC4620412; doi:10.3389/fpls.2015.00857)
Supplement: Supplementary file 1 [file DataSheet1.PDF]

Species identity and neighbor size surpass the impact of tree species diversity on productivity in experimental broad-leaved tree sapling assemblages under dry and moist conditions

Supplemental material

Torben Lübbe[1*], Bernhard Schuldt[1] and Christoph Leuschner[1]

[1] *Plant Ecology and Ecosystems Research, Albrecht von Haller Institute for Plant Sciences, University of Göttingen*

[*] ***Correspondence:*** *Torben Lübbe, Albrecht von Haller Institute for Plant Sciences, Ecology and Ecosystem Research, University of Goettingen, Grisebachstr. 1, 37077 Goettingen, Germany. E-mail: tluebbe@gwdg.de, Phone: +49-(0)551-39-5702.*

Table A1: Initial size and biomass of the tree saplings of the five species used in the experiment (mean ± SE, n=12): Phytomass, root-shoot ratio (RS), basal area (BA), shoot length and root length. Different small letters indicate significant differences among species ($p<0.05$).

Species	Phytomass [g]	RS [g g^{-1}]	BA [cm^2]	L_{Shoot} [cm]	L_{Root} [cm]
A. pseudopl.	16.92 ± 0.89 a	1.21 ± 0.05 a	0.30 ± 0.00 a	61.76 ± 2.92 c	20.05 ± 1.13 a
C. betulus	20.41 ± 1.54 ab	1.35 ± 0.07 ab	0.38 ± 0.01 a	54.16 ± 2.42 bc	20.32 ± 0.94 a
F. sylvatica	16.20 ± 0.43 a	1.59 ± 0.06 cd	0.27 ± 0.00 a	36.90 ± 1.70 a	27.27 ± 1.62 b
F. excelsior	20.27 ± 1.10 ab	1.48 ± 0.03 bc	0.50 ± 0.01 a	44.46 ± 1.12 ab	22.52 ± 1.18 ab
T. cordata	21.43 ± 1.29 b	1.75 ± 0.07 d	1.24 ± 0.01 b	40.09 ± 1.77 a	35.18 ± 1.67 c

Table A2: Parameters characterizing productivity and plant-internal biomass partitioning at the level of the plant individual for the five species in the moist and dry treatment (means ± SE). Data are averages over all species combinations to show species differences (n = 57 and 32 pots in the moist and dry treatment, respectively). Relative growth rates (RGR) are given in g g^{-1}450 d^{-1}. Different capital letters indicate significant differences among species (p<0.05) in the moist or dry treatment. Asterisks indicate significant differences among the treatments (°: p<0.10; *: p<0.05; **: p<0.01; ***: p<0.001).

Species	Treatment	Phytomass [g]	RS [g g^{-1}]	LA [m^2]	BA [cm^2]
F. excelsior	moist	147.20 ± 8.60 C	1.09 ± 0.04 B	0.36 ± 0.02 B	2.47 ± 0.14 C
A. pseudopl.	moist	68.22 ± 3.85 A	1.45 ± 0.06 C	0.17 ± 0.01 A	1.21 ± 0.05 A
C. betulus	moist	99.87 ± 4.79 B	0.73 ± 0.02 A	0.38 ± 0.02 B	1.81 ± 0.07 B
T. cordata	moist	161.49 ± 8.10 C	1.08 ± 0.04 B	0.53 ± 0.02 C	4.51 ± 0.19 D
F. sylvatica	moist	77.70 ± 3.83 AB	1.09 ± 0.03 B	0.21 ± 0.01 A	1.30 ± 0.05 A
F. excelsior	dry	121.05 ± 6.66 C *	1.14 ± 0.03 B	0.29 ± 0.02 B °	1.87 ± 0.09 C **
A. pseudopl.	dry	54.28 ± 3.75 A *	1.41 ± 0.06 C	0.15 ± 0.01 A	0.97 ± 0.06 A **
C. betulus	dry	82.57 ± 5.24 B *	0.73 ± 0.02 A	0.34 ± 0.02 B	1.55 ± 0.08 BC *
T. cordata	dry	115.53 ± 6.14 C ***	1.02 ± 0.04 B	0.43 ± 0.03 C *	3.44 ± 0.16 D ***
F. sylvatica	dry	64.20 ± 3.34 AB *	1.04 ± 0.04 B	0.20 ± 0.01 A	1.20 ± 0.06 AB

Species	Treatment	L_{Shoot} [cm]	L_{Root} [cm]	LI_{Shoot} [%]	LI_{Root} [%]
F. excelsior	moist	127.66 ± 4.34 D	68.75 ± 1.81 B	187.12 ± 9.77 D	205.28 ± 8.04 B
A. pseudopl.	moist	73.53 ± 2.60 A	81.23 ± 2.54 C	34.90 ± 3.83 A	305.10 ± 12.67 C
C. betulus	moist	109.43 ± 2.50 C	58.06 ± 1.30 A	103.13 ± 4.37 B	185.72 ± 6.40 B
T. cordata	moist	102.33 ± 2.43 C	108.40 ± 2.38 D	156.26 ± 5.92 C	208.14 ± 6.77 B
F. sylvatica	moist	88.43 ± 2.50 B	68.48 ± 1.86 B	139.64 ± 6.78 C	151.12 ± 6.81 A
F. excelsior	dry	101.82 ± 3.10 B ***	64.13 ± 1.77 B °	129.02 ± 6.96 C ***	184.76 ± 7.84 C °
A. pseudopl.	dry	68.19 ± 2.83 A	74.16 ± 2.50 C °	23.99 ± 4.00 A	269.83 ± 12.47 D °
C. betulus	dry	97.04 ± 2.50 B **	48.95 ± 1.24 A ***	79.19 ± 4.61 B ***	140.90 ± 6.12 B ***
T. cordata	dry	94.28 ± 3.89 B *	86.02 ± 2.38 D ***	135.17 ± 9.70 C *	144.51 ± 6.76 B ***
F. sylvatica	dry	72.42 ± 2.26 A ***	56.87 ± 1.81 B ***	96.25 ± 6.14 B ***	108.53 ± 6.65 A ***

Species	Treatment	BAI [%]	RGR_{above}	RGR_{below}	RGR_{total}
F. excelsior	moist	399.30 ± 29.23 C	8.60 ± 0.63 D	5.77 ± 0.40 B	6.92 ± 0.46 B
A. pseudopl.	moist	307.86 ± 17.66 AB	2.92 ± 0.25 A	3.42 ± 0.25 A	3.18 ± 0.24 A
C. betulus	moist	378.52 ± 18.70 BC	6.67 ± 0.40 C	3.22 ± 0.19 A	4.71 ± 0.26 A
T. cordata	moist	264.90 ± 15.22 A	9.47 ± 0.55 D	5.35 ± 0.32 B	6.78 ± 0.39 B
F. sylvatica	moist	383.85 ± 18.03 BC	5.01 ± 0.32 B	3.02 ± 0.19 A	3.80 ± 0.24 A
F. excelsior	dry	277.65 ± 18.68 BC **	6.56 ± 0.44 C °	4.81 ± 0.33 C °	5.52 ± 0.36 C *
A. pseudopl.	dry	228.11 ± 21.33 AB **	2.16 ± 0.29 A °	2.48 ± 0.21 A *	2.33 ± 0.23 A *
C. betulus	dry	311.40 ± 21.63 C *	5.22 ± 0.40 B *	2.47 ± 0.22 A **	3.67 ± 0.30 B *
T. cordata	dry	182.74 ± 12.39 A ***	6.95 ± 0.50 C **	3.41 ± 0.19 B ***	4.64 ± 0.29 C ***
F. sylvatica	dry	347.52 ± 20.89 C	4.07 ± 0.30 B °	2.29 ± 0.16 A *	2.99 ± 0.21 AB *

Table A3: Additive partitioning of biodiversity effects on productivity as measured by various growth-related parameters: above- and belowground biomass (Bm), leaf area (LA), basal area (BA), shoot and root length (L). Asterisks indicate the significance levels of the effect size (grand mean, t-test) and the significance of species richness (3 vs. 5 species; Wilcoxon-test) or species composition influences on the three effects (ANOVA; *: p<0.05; **: p<0.01; ***: p<0.001).

		Moist treatment			Dry treatment		
Parameter		Net effect	Selection	Complementarity	Net effect	Selection	Complementarity
Bm_{Above}	Grand mean	**		**	*		*
	Richness						
	Composition		***			*	
Bm_{Below}	Grand mean	**	*	**			
	Richness						
	Composition		**				
LA	Grand mean	***	*	***	**		***
	Richness						
	Composition		***				
BA	Grand mean	**	**	**			
	Richness						
	Composition						
L_{Shoot}	Grand mean						
	Richness						
	Composition		**				
L_{Root}	Grand mean	***		***			
	Richness						
	Composition		*				

Table A4: Growth performance of *Fraxinus excelsior* in the moist and dry treatments and the three diversity levels as measured by 12 productivity and biomass partitioning parameters (means ± SE). Relative growth rates (RGR) are given in g g^{-1}450 d^{-1}. Different small letters indicate significant differences between the diversity levels (p<0.05) in the moist or dry treatment. Asterisks indicate significant differences among the treatments (°: p<0.10; *: p<0.05; **: p<0.01; ***: p<0.001).

Moisture treatment	Diversity level	Sample size [n]	Phytomass [g]	RS [g g^{-1}]	LA [m^2]	BA [cm^2]
moist	mono	7	135.93 ± 16.48 a	0.97 ± 0.08 a	0.34 ± 0.04 a	2.08 ± 0.21 a
moist	mix 3	41	153.87 ± 10.68 a	1.12 ± 0.05 a	0.38 ± 0.03 a	2.58 ± 0.18 a
moist	mix 5	7	119.38 ± 17.70 a	1.07 ± 0.09 a	0.27 ± 0.05 a	2.25 ± 0.33 a
dry	mono	7	116.25 ± 6.46 a	1.18 ± 0.05 a **	0.25 ± 0.01 a *	1.78 ± 0.16 a
dry	mix 3	17	122.05 ± 9.60 a *	1.14 ± 0.05 a	0.30 ± 0.03 a *	1.84 ± 0.09 a **
dry	mix 5	7	123.43 ± 18.38 a	1.10 ± 0.09 a	0.29 ± 0.04 a	2.03 ± 0.33 a

Moisture treatment	Diversity level	Sample size [n]	L_{Shoot} [cm]	L_{Root} [cm]	LI_{Shoot} [%]	LI_{Root} [%]
moist	mono	7	131.26 ± 6.93 a	60.37 ± 4.01 a	195.25 ± 15.59 a	168.20 ± 17.84 a
moist	mix 3	41	126.35 ± 5.39 a	69.10 ± 2.11 a	184.15 ± 12.13 a	206.80 ± 9.38 a
moist	mix 5	7	132.45 ± 12.86 a	75.09 ± 4.76 a	197.92 ± 28.93 a	233.47 ± 21.13 a
dry	mono	7	104.21 ± 2.07 a **	60.41 ± 2.27 a	134.39 ± 4.65 a *	168.37 ± 10.10 a
dry	mix 3	17	98.48 ± 4.46 a ***	64.44 ± 2.41 a	121.50 ± 10.02 a ***	186.12 ± 10.71 a
dry	mix 5	7	107.54 ± 8.27 a	67.07 ± 4.74 a	141.90 ± 18.61 a	197.88 ± 21.07 a

Moisture treatment	Diversity level	Sample size [n]	BAI [%]	RGR_{above}	RGR_{below}	RGR_{total}
moist	mono	7	320.34 ± 41.95 a	8.38 ± 0.70 a	4.91 ± 0.78 a	6.32 ± 0.89 a
moist	mix 3	41	420.38 ± 36.68 a	8.95 ± 0.79 a	6.15 ± 0.50 a	7.28 ± 0.57 a
moist	mix 5	7	354.78 ± 65.71 a	6.83 ± 1.30 a	4.47 ± 0.79 a	5.43 ± 0.95 a
dry	mono	7	259.07 ± 32.47 a	6.08 ± 0.36 a *	4.69 ± 0.37 a	5.26 ± 0.35 a
dry	mix 3	17	271.64 ± 17.22 a **	6.56 ± 0.61 a *	4.89 ± 0.49 a °	5.57 ± 0.52 a *
dry	mix 5	7	310.81 ± 67.05 a	7.01 ± 1.32 a	4.71 ± 0.80 a	5.64 ± 0.99 a

Table A5: Growth performance of *Acer pseudoplatanus* in the moist and dry treatments and the three diversity levels as measured by 12 productivity and biomass partitioning parameters (means ± SE). Relative growth rates (RGR) are given in g g^{-1}450 d^{-1}. Different small letters indicate significant differences between the diversity levels (p<0.05) in the moist or dry treatment. Asterisks indicate significant differences among the treatments (°: p<0.10; *: p<0.05; **: p<0.01; ***: p<0.001).

Moisture treatment	Diversity level	Sample size [n]	Phytomass [g]	RS [g g^{-1}]	LA [m^2]	BA [cm^2]
moist	mono	7	76.58 ± 9.40 a	1.57 ± 0.19 a	0.19 ± 0.02 a	1.28 ± 0.14 a
moist	mix 3	40	67.84 ± 4.62 a	1.44 ± 0.06 a	0.17 ± 0.01 a	1.19 ± 0.06 a
moist	mix 5	8	62.76 ± 10.55 a	1.43 ± 0.18 a	0.14 ± 0.02 a	1.21 ± 0.12 a
dry	mono	7	58.51 ± 6.31 a	1.63 ± 0.13 b	0.15 ± 0.02 a	0.98 ± 0.12 a
dry	mix 3	15	48.82 ± 3.59 a *	1.45 ± 0.05 b	0.13 ± 0.01 a *	0.89 ± 0.05 a *
dry	mix 5	7	61.76 ± 12.05 a	1.10 ± 0.11 a	0.18 ± 0.04 a	1.11 ± 0.21 a

Moisture treatment	Diversity level	Sample size [n]	L_{Shoot} [cm]	L_{Root} [cm]	LI_{Shoot} [%]	LI_{Root} [%]
moist	mono	7	72.55 ± 6.17 a	68.59 ± 2.01 a	24.50 ± 8.77 a	242.05 ± 10.00 a
moist	mix 3	40	73.33 ± 2.70 a	81.20 ± 2.97 ab	34.40 ± 3.95 a	304.92 ± 14.82 ab
moist	mix 5	8	75.39 ± 11.20 a	92.46 ± 7.45 b	57.10 ± 18.87 a	361.16 ± 37.16 b
dry	mono	7	67.88 ± 4.03 a	70.04 ± 3.69 a	23.15 ± 4.40 a	249.38 ± 18.43 a
dry	mix 3	15	63.48 ± 2.34 a **	73.29 ± 2.76 a	13.89 ± 2.06 a **	265.45 ± 13.78 a
dry	mix 5	7	78.59 ± 9.24 a	80.13 ± 7.75 a	43.17 ± 13.18 a	299.64 ± 38.65 a

Moisture treatment	Diversity level	Sample size [n]	BAI [%]	RGR_{above}	RGR_{below}	RGR_{total}
moist	mono	7	333.02 ± 47.50 a	3.25 ± 0.70 a	4.07 ± 0.52 a	3.70 ± 0.58 a
moist	mix 3	40	303.38 ± 21.66 a	2.93 ± 0.29 a	3.39 ± 0.31 a	3.16 ± 0.28 a
moist	mix 5	8	308.26 ± 41.04 a	2.63 ± 0.79 a	3.03 ± 0.60 a	2.85 ± 0.65 a
dry	mono	7	231.18 ± 39.96 a	2.16 ± 0.41 a	2.98 ± 0.41 a	2.59 ± 0.39 a
dry	mix 3	15	201.66 ± 17.53 a *	1.69 ± 0.20 a *	2.25 ± 0.25 a °	1.99 ± 0.22 a *
dry	mix 5	7	277.32 ± 71.68 a	3.16 ± 1.00 a	2.47 ± 0.54 a	2.79 ± 0.74 a

Table A6: Growth performance of *Carpinus betulus* in the moist and dry treatments and the three diversity levels as measured by 12 productivity and biomass partitioning parameters (means ± SE). Relative growth rates (RGR) are given in g g^{-1}450 d^{-1}. Different small letters indicate significant differences between the diversity levels ($p<0.05$) in the moist or dry treatment. Asterisks indicate significant differences among the treatments (°: $p<0.10$; *: $p<0.05$; **: $p<0.01$; ***: $p<0.001$).

Moisture treatment	Diversity level	Sample size [n]	Phytomass [g]	RS [g g^{-1}]	LA [m^2]	BA [cm^2]
moist	mono	7	88.06 ± 9.48 a	0.81 ± 0.03 a	0.33 ± 0.02 a	1.64 ± 0.10 a
moist	mix 3	41	100.94 ± 5.74 a	0.71 ± 0.02 a	0.39 ± 0.02 a	1.80 ± 0.08 a
moist	mix 5	7	105.41 ± 14.66 a	0.78 ± 0.05 a	0.40 ± 0.06 a	2.04 ± 0.24 a
dry	mono	7	72.11 ± 4.55 a	0.71 ± 0.03 a *	0.31 ± 0.01 a	1.38 ± 0.05 a °
dry	mix 3	16	85.56 ± 8.99 a °	0.72 ± 0.03 a	0.35 ± 0.03 a	1.65 ± 0.14 a °
dry	mix 5	7	86.19 ± 7.96 a	0.76 ± 0.04 a	0.37 ± 0.03 a	1.51 ± 0.12 a

Moisture treatment	Diversity level	Sample size [n]	L_{Shoot} [cm]	L_{Root} [cm]	LI_{Shoot} [%]	LI_{Root} [%]
moist	mono	7	108.49 ± 4.59 a	58.27 ± 3.81 a	100.31 ± 8.47 a	186.83 ± 18.76 a
moist	mix 3	41	110.41 ± 3.12 a	57.43 ± 1.50 a	105.32 ± 5.39 a	182.60 ± 7.41 a
moist	mix 5	7	104.63 ± 6.03 a	61.54 ± 3.76 a	93.19 ± 11.12 a	202.92 ± 18.53 a
dry	mono	7	92.39 ± 4.05 a *	48.67 ± 1.69 a *	70.59 ± 7.48 a *	139.51 ± 8.33 a *
dry	mix 3	16	99.54 ± 3.21 a *	48.58 ± 1.59 a ***	83.80 ± 5.94 a *	139.01 ± 7.81 a ***
dry	mix 5	7	95.99 ± 6.88 a	50.10 ± 3.81 a *	77.23 ± 12.71 a	146.60 ± 18.73 a *

Moisture treatment	Diversity level	Sample size [n]	BAI [%]	RGR_{above}	RGR_{below}	RGR_{total}
moist	mono	7	333.98 ± 27.66 a	5.29 ± 0.59 a	3.09 ± 0.46 a	3.98 ± 0.54 a
moist	mix 3	41	375.46 ± 22.07 a	6.91 ± 0.50 a	3.16 ± 0.20 a	4.79 ± 0.31 a
moist	mix 5	7	440.98 ± 62.87 a	6.61 ± 0.99 a	3.69 ± 0.74 a	4.97 ± 0.83 a
dry	mono	7	266.17 ± 14.30 a °	4.47 ± 0.28 a	2.04 ± 0.22 a °	3.08 ± 0.26 a
dry	mix 3	16	336.11 ± 37.22 a	5.49 ± 0.69 a °	2.56 ± 0.38 a *	3.99 ± 0.52 a
dry	mix 5	7	300.15 ± 31.24 a °	5.37 ± 0.64 a	2.72 ± 0.32 a	3.88 ± 0.45 a

Table A7: Growth performance of *Tilia cordata* in the moist and dry treatments and the three diversity levels as measured by 12 productivity and biomass partitioning parameters (means ± SE). Relative growth rates (RGR) are given in g g^{-1}450 d^{-1}. Different small letters indicate significant differences between the diversity levels ($p<0.05$) in the moist or dry treatment. Asterisks indicate significant differences among the treatments (°: $p<0.10$; *: $p<0.05$; **: $p<0.01$; ***: $p<0.001$).

Moisture treatment	Diversity level	Sample size [n]	Phytomass [g]	RS [g g^{-1}]	LA [m^2]	BA [cm^2]
moist	mono	7	122.01 ± 6.20 a	1.12 ± 0.09 a	0.34 ± 0.02 a	3.73 ± 0.13 a
moist	mix 3	40	160.66 ± 9.60 ab	1.09 ± 0.05 a	0.55 ± 0.03 b	4.51 ± 0.23 a
moist	mix 5	8	200.17 ± 21.80 b	1.03 ± 0.09 a	0.61 ± 0.04 b	5.23 ± 0.45 a
dry	mono	7	107.26 ± 4.40 a °	1.01 ± 0.04 a	0.37 ± 0.01 a	3.16 ± 0.12 a **
dry	mix 3	15	121.67 ± 10.25 a *	0.99 ± 0.06 a	0.48 ± 0.05 a	3.59 ± 0.27 a *
dry	mix 5	7	110.67 ± 12.43 a **	1.07 ± 0.08 a	0.41 ± 0.05 a **	3.42 ± 0.28 a **

Moisture treatment	Diversity level	Sample size [n]	L_{Shoot} [cm]	L_{Root} [cm]	LI_{Shoot} [%]	LI_{Root} [%]
moist	mono	7	92.60 ± 3.70 a	104.03 ± 5.11 a	138.85 ± 3.62 a	195.79 ± 14.52 a
moist	mix 3	40	102.29 ± 3.01 a	108.20 ± 2.80 a	155.13 ± 7.50 a	207.57 ± 7.98 a
moist	mix 5	8	111.09 ± 5.40 a	113.20 ± 7.52 a	177.08 ± 13.46 a	221.82 ± 21.37 a
dry	mono	7	93.74 ± 1.49 a	86.19 ± 3.60 a *	133.82 ± 3.71 a	144.97 ± 10.21 a *
dry	mix 3	15	98.12 ± 6.52 a	83.30 ± 2.61 a ***	144.75 ± 16.27 a	136.77 ± 7.43 a ***
dry	mix 5	7	86.60 ± 7.88 a *	91.69 ± 7.37 a °	116.00 ± 19.66 a *	160.66 ± 20.96 a °

Moisture treatment	Diversity level	Sample size [n]	BAI [%]	RGR_{above}	RGR_{below}	RGR_{total}
moist	mono	7	209.49 ± 11.26 a	7.02 ± 0.40 a	4.07 ± 0.38 a	5.05 ± 0.37 a
moist	mix 3	40	264.42 ± 18.88 a	9.39 ± 0.67 ab	5.29 ± 0.37 a	6.72 ± 0.46 ab
moist	mix 5	8	322.42 ± 36.12 a	11.97 ± 1.21 b	6.70 ± 1.04 a	8.61 ± 1.05 b
dry	mono	7	155.66 ± 10.05 a **	6.14 ± 0.27 a	3.02 ± 0.21 a °	4.15 ± 0.21 a °
dry	mix 3	15	190.01 ± 22.14 a *	7.66 ± 0.82 a	3.68 ± 0.30 a **	5.15 ± 0.47 a *
dry	mix 5	7	176.51 ± 22.99 a **	6.23 ± 0.99 a **	3.22 ± 0.40 a *	4.32 ± 0.60 a **

Table A8: Growth performance of *Fagus sylvatica* in the moist and dry treatments and the three diversity levels as measured by 12 productivity and biomass partitioning parameters (means ± SE). Relative growth rates (RGR) are given in g g^{-1}450 d^{-1}. Different small letters indicate significant differences between the diversity levels ($p<0.05$) in the moist or dry treatment. Asterisks indicate significant differences among the treatments (°: $p<0.10$; *: $p<0.05$; **: $p<0.01$; ***: $p<0.001$).

Moisture treatment	Diversity level	Sample size [n]	Phytomass [g]	RS [g g^{-1}]	LA [m^2]	BA [cm^2]
moist	mono	7	94.67 ± 11.24 a	0.90 ± 0.06 a	0.28 ± 0.03 b	1.62 ± 0.13 b
moist	mix 3	40	71.87 ± 3.55 a	1.09 ± 0.03 b	0.20 ± 0.01 a	1.21 ± 0.05 a
moist	mix 5	8	91.99 ± 15.57 a	1.21 ± 0.07 b	0.21 ± 0.03 ab	1.46 ± 0.18 ab
dry	mono	7	71.58 ± 6.49 a	1.03 ± 0.07 a	0.21 ± 0.02 a °	1.29 ± 0.06 a *
dry	mix 3	17	61.47 ± 4.20 a °	1.05 ± 0.04 a	0.20 ± 0.01 a	1.13 ± 0.08 a
dry	mix 5	7	63.44 ± 8.85 a °	1.05 ± 0.12 a	0.19 ± 0.02 a	1.28 ± 0.13 a

Moisture treatment	Diversity level	Sample size [n]	L_{Shoot} [cm]	L_{Root} [cm]	LI_{Shoot} [%]	LI_{Root} [%]
moist	mono	7	98.61 ± 7.48 a	64.81 ± 2.67 a	167.22 ± 20.27 a	137.74 ± 9.79 a
moist	mix 3	40	87.26 ± 2.86 a	67.23 ± 2.21 a	136.48 ± 7.75 a	146.50 ± 8.09 a
moist	mix 5	8	85.35 ± 6.86 a	77.80 ± 5.19 a	131.30 ± 18.58 a	185.33 ± 19.03 a
dry	mono	7	74.90 ± 2.80 a *	57.97 ± 2.18 a °	102.99 ± 7.60 a *	112.64 ± 7.99 a °
dry	mix 3	17	71.90 ± 3.24 a ***	55.69 ± 2.63 a **	94.84 ± 8.77 a ***	104.18 ± 9.64 a **
dry	mix 5	7	71.19 ± 6.03 a	58.61 ± 4.70 a *	92.91 ± 16.35 a	114.97 ± 17.22 a *

Moisture treatment	Diversity level	Sample size [n]	BAI [%]	RGR_{above}	RGR_{below}	RGR_{total}
moist	mono	7	502.51 ± 48.29 b	7.09 ± 0.99 b	3.40 ± 0.55 a	4.84 ± 0.69 a
moist	mix 3	40	351.51 ± 17.16 a	4.50 ± 0.29 a	2.75 ± 0.18 a	3.44 ± 0.22 a
moist	mix 5	8	441.71 ± 65.65 ab	5.75 ± 1.30 ab	3.99 ± 0.76 a	4.68 ± 0.96 a
dry	mono	7	381.52 ± 21.02 a *	4.67 ± 0.58 a °	2.78 ± 0.28 a	3.61 ± 0.35 a
dry	mix 3	17	321.76 ± 30.82 a	3.80 ± 0.36 a	2.15 ± 0.21 a *	2.71 ± 0.26 a *
dry	mix 5	7	376.09 ± 49.76 a	4.10 ± 0.86 a	2.15 ± 0.39 a *	2.92 ± 0.55 a

Table A9: Pot-level water use efficiency (WUE) of tree assemblages differing in species composition and diversity in the moist and dry treatments (means ± SE, for no. of replicates see Table 1). WUE is given as total biomass per plant produced in the 450 d-experiment divided by the mean daily transpiration in June 2012 at peak transpiration (data after Lübbe et al., 2015). Different small letters indicate significant differences in WUE of the species or species combinations in the monospecific or mix 3 groups, or between the diversity levels. Asterisks in the dry treatment column mark significantly higher WUE in the dry than the moist treatment (*: p<0.05; **: p<0.01; ***: p<0.001).

Diversity level / Species composition	WUE [(g * 450 d^{-1})/(ml * d^{-1})] Moist	Dry	
mono			
F. excelsior	0.58 ± 0.30 a	0.77 ± 0.13 b	*
A. pseudoplatanus	0.52 ± 0.47 a	0.51 ± 0.13 a	
C. betulus	0.44 ± 0.30 a	0.65 ± 0.16 ab	*
T. cordata	0.49 ± 0.31 a	0.64 ± 0.13 ab	*
F. sylvatica	0.47 ± 0.34 a	0.69 ± 0.15 ab	**
mix 3			
A.p. - C.b. - F.e.	0.54 ± 0.18 a	0.61 ± 0.07 a	
A.p. - C.b. - T.c.	0.48 ± 0.35 a	0.59 ± 0.14 a	
A.p. - F.s. - F.e.	0.54 ± 0.43 a	0.64 ± 0.07 a	
C.b. - F.s. - T.c.	0.49 ± 0.27 a	0.64 ± 0.11 a	*
F.s. - F.e. - T.c.	0.53 ± 0.20 a	0.67 ± 0.09 a	*
Diversity level			
mono	0.50 ± 0.13 a	0.65 ± 0.15 a	***
mix 3	0.52 ± 0.10 a	0.63 ± 0.10 a	***
mix 5	0.51 ± 0.13 a	0.63 ± 0.11 a	*

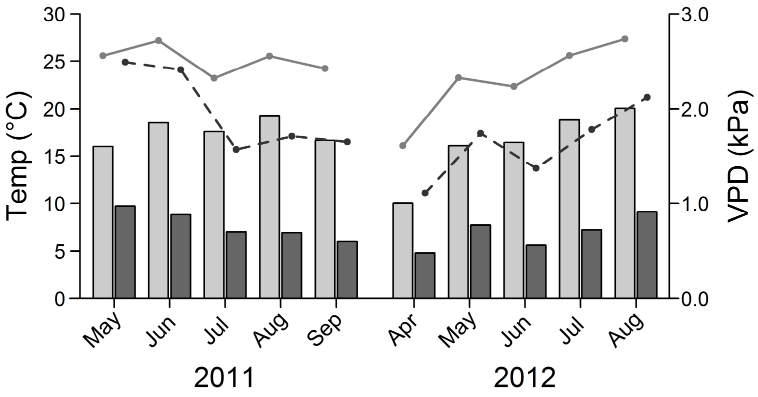

Figure A1: Temperature (bright bars, solid line) and vapor pressure deficit (dark bars, dashed line) at the experimental site in the summers of 2011 and 2012 (means per month). Lines give monthly means of daily maxima.

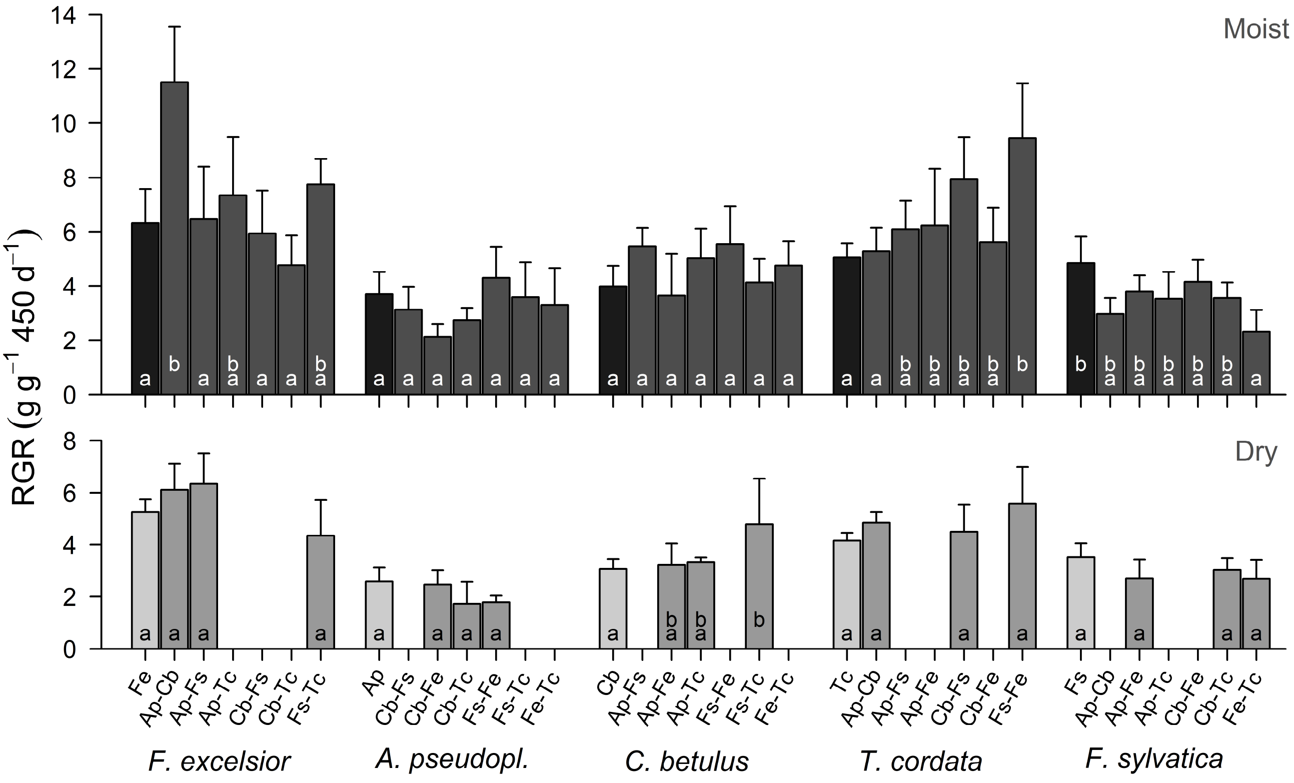

Figure A2: Relative growth rate of the five species either in monoculture (first bar in a bloc) or in the six different 3-species combinations in the moist (upper panel) and dry treatment (lower panel) (means ± SE of 7 or 6 replicate pots). In the dry treatment, only three 3-species combinations were realized. For species abbreviations see Table 1. Different letters indicate significant differences within a species ($p<0.05$).
